# Feasibility of a drop-in γ-probe for radioguided sentinel lymph detection in early-stage cervical cancer

**DOI:** 10.1186/s13550-022-00907-w

**Published:** 2022-06-20

**Authors:** Ilse G. T. Baeten, Jacob P. Hoogendam, Arthur J. A. T. Braat, Ronald P. Zweemer, Cornelis G. Gerestein

**Affiliations:** 1 Division of Imaging and Oncology, Department of Gynecologic Oncology, University Medical Center Utrecht, Utrecht University, Heidelberglaan 100, 3584 CX Utrecht, The Netherlands; 2 Division of Imaging and Oncology, Department of Radiology and Nuclear Medicine, University Medical Center Utrecht, Utrecht University, Utrecht, The Netherlands

**Keywords:** Radioguided surgery, Gamma probe, Sentinel lymph node, Cervical cancer, Robot-assisted surgery

## Abstract

**Background:**

Minimally invasive radioguided sentinel lymph node (SLN) procedures, increasingly performed with robot-assisted laparoscopy, can benefit from using a drop-in γ-probe instead of the conventional rigid laparoscopic γ-probe. We evaluated the safety and feasibility of a tethered drop-in γ-probe system for SLN detection in patients with early-stage cervical cancer.

**Methods:**

Ten patients with FIGO stage IA – IB2 or IIA1 cervical cancer scheduled for robot-assisted laparoscopic SLN procedure were included. All patients underwent preoperative 240 MBq technetium-99m nanocolloid (^99m^Tc) injection and SPECT/CT imaging. Intraoperatively the tethered drop-in γ-probe SENSEI® (Lightpoint Medical Ltd, Chesham, UK) was used for probe guided SLN detection, subsequently confirmed by the standard rigid laparoscopic γ-probe. Sentinel lymph node detection rates and anatomical SLN location were assessed. Surgeon questionnaires were used to assess usability.

**Results:**

In all patients at least one SLN was successfully resected under guidance of the drop-in γ-probe (overall detection rate: 100%). Bilateral SLN detection rate with the drop-in γ-probe was 80%. Of the two patients with unilateral SLN detection only, one presented with an atypical SLN location at the aortic bifurcation that was detected only on SPECT/CT. The other patient had failed unilateral ^99m^Tc uptake. Combined use of preoperative SPECT/CT and drop-in γ-probe resulted in a bilateral detection rate of 90%. Similar to the drop-in γ-probe, overall and bilateral SLN detection rate of the rigid γ-probe was 100% and 80%, respectively. No significant discrepancy existed between the count rate of the drop-in and rigid laparoscopic γ-probe (*p* = 0.69). In total 21 SLN’s were detected with the drop-in γ-probes including all three tumor positive nodes. Because of wristed articulation of the robotic tissue grasper and possibility of autonomous probe control by the surgeon, maneuverability and control with the drop-in γ-probe were highly rated in surgeon questionnaires. No adverse events related to the intervention occurred.

**Conclusions:**

Sentinel lymph node detection with a drop-in γ-probe is safe and feasible in patients with early-stage cervical cancer. Use of the drop-in γ-probe enhances maneuverability and surgical autonomy during robot-assisted SLN detection.

*Trial registration* Netherlands Trial Registry, NL9358. Registered 23 March 2021, https://www.trialregister.nl/trial/9358.

**Supplementary Information:**

The online version contains supplementary material available at 10.1186/s13550-022-00907-w.

## Background

The concept of sentinel lymph node (SLN) procedure is based on the existence of an orderly and predictable pattern of lymphatic drainage, with the first lymph node functioning as an effective filter for tumor cells that can be detected with a tracer or dye. Identifying SLNs by using the gamma emitting potential of radionuclides was a technology first introduced in the nineties [1, 2], and it has been the most researched technology for SLN detection in cervical cancer ever since [3–5]. The most frequently used radiopharmaceutical, technetium-99m nanocolloid (^99m^Tc), enables both preoperative imaging with SPECT/CT and intraoperative guidance toward SLNs with a γ-probe. The shift toward minimally invasive surgery, including SLN detection to be a laparoscopic and robot-assisted procedure, has resulted in the development of rigid laparoscopic (elongated) γ-probes that can be inserted through a trocar. Application of these conventional rigid γ-probes during laparoscopic or robot-assisted surgery has several disadvantages. Their long rigid design and insertion through a trocar restricts the rotational freedom and maneuverability during surgery. Use of a rigid laparoscopic γ-probe can restrict reaching anatomical locations deep in the pelvic retroperitoneal space under a desired angle. Furthermore, when radioactive SLNs are located near a high activity background (i.e. point of tracer injection), careful probe positioning becomes pivotal in order to avoid background noise with falsely elevated measurements [6, 7]. With implementation of robot-assisted surgery, rigid probe positioning is further complicated as the surgeon no longer directly guides the probe himself, but now verbally guides the bedside assistant to handle the rigid laparoscopic γ-probe. Between robotic arms, accessibility of the trocar may be difficult and can further restrict maneuverability. A small laparoscopic tethered drop-in γ-probe (TDIP) which can be released in the abdominal cavity and grasped by the surgeon with a (robotic) laparoscopic forceps could overcome these drawbacks, as previously showed in prostate cancer patients [8, 9].

This first-in-women pilot study aims to assess the feasibility, specifically the performance and anticipated technical advantages in terms of SLN detection rate, of TDIP during robot-assisted SLN procedures in cervical cancer patients.

## Methods

### Patient population and design

A prospective, single-center, single-arm feasibility study included adult female patients with histologically proven cervical cancer FIGO stage IA – IB2 or IIA1 (TNM: T1a1 – T1b2 or T2a1) scheduled for radical robot-assisted surgery including SLN procedure [10]. The patient was admitted one day preoperatively to undergo preoperative SPECT/CT imaging. The following day robot-assisted surgery including SLN procedure was performed. Standard medical treatment and follow-up was performed according to international guidelines [11] The study was prospectively registered in the Netherlands Trial Registry (NL9358).

Main outcome was the feasibility of a TDIP assessed by SLN detection rate. Overall SLN detection rate was defined as in vivo detection of at least one SLN in each patient whereas bilateral detection rate was defined as in vivo detection of at least one SLN in each hemipelvis. Secondary outcomes included number of SLNs detected, correlation with preoperative imaging (SPECT/CT), intraoperative adverse events, and overall ease of use (assessed by a questionnaire filled in by surgeons).

In total ten patients were included to assess feasibility. Clinical, surgical and pathological baseline characteristics of included patients were collected.

### Preoperative imaging

One day prior to surgery (with an 18–20 h interval to surgery), a dose of 240 MBq ^99m^Tc-nanocolloid was injected into four quadrants of the cervix (60 MBq into each quadrant) by an experienced gynecological oncologist (according to the current standard of care). Ninety minutes post injection, anterior and posterior static planar imaging (4 min) was performed of the lower abdomen. Subsequently, a SPECT/CT (Symbia T16, Siemens, Erlangen, Germany) was performed (128 × 128 matrix, step-and-shoot, 20 s/view, 64 views) with a contrast enhanced abdominal CT (with a patient dependent current/mAs, 110 kV, aortic triggered 100 HU with 50 s delay). The SPECT/CT was reviewed by nuclear physicians preoperatively resulting in a written report including a detailed description of the injection procedure, ^99m^Tc-nanocolloid dosing, imaging procedure, and number and anatomical location of all visualized SLNs according to a pre-specified format (see Additional file 1).

### Surgical procedure

Two gynecological oncologists specialized in robot-assisted surgery (RZ and CG; both more than five years of experience) performed all procedures using a four-armed surgical robotic da Vinci® system (*X* or *X*i, Intuitive Surgical, Sunnyvale, USA). The surgical procedure included a SLN procedure, pelvic lymph node dissection and, in case of negative SLNs at frozen section examination, a radical hysterectomy or radical vaginal trachelectomy. Details of the surgical techniques performed have been previously described [12]. Intraoperatively, patent blue dye was used to help identify SLNs (according to current standard of care). A proportion of patients (*n* = 9) also received indocyanine green (ICG) for their participation in the FluoreSENT study (Netherlands Trial Registry NL9011). These dyes were consecutively injected into four quadrants of the cervix, identical to the ^99m^Tc injection, directly prior to SLN detection. Sentinel lymph node detection with ICG and/or blue dye is not within the scope of this manuscript.

**Fig. 1:**
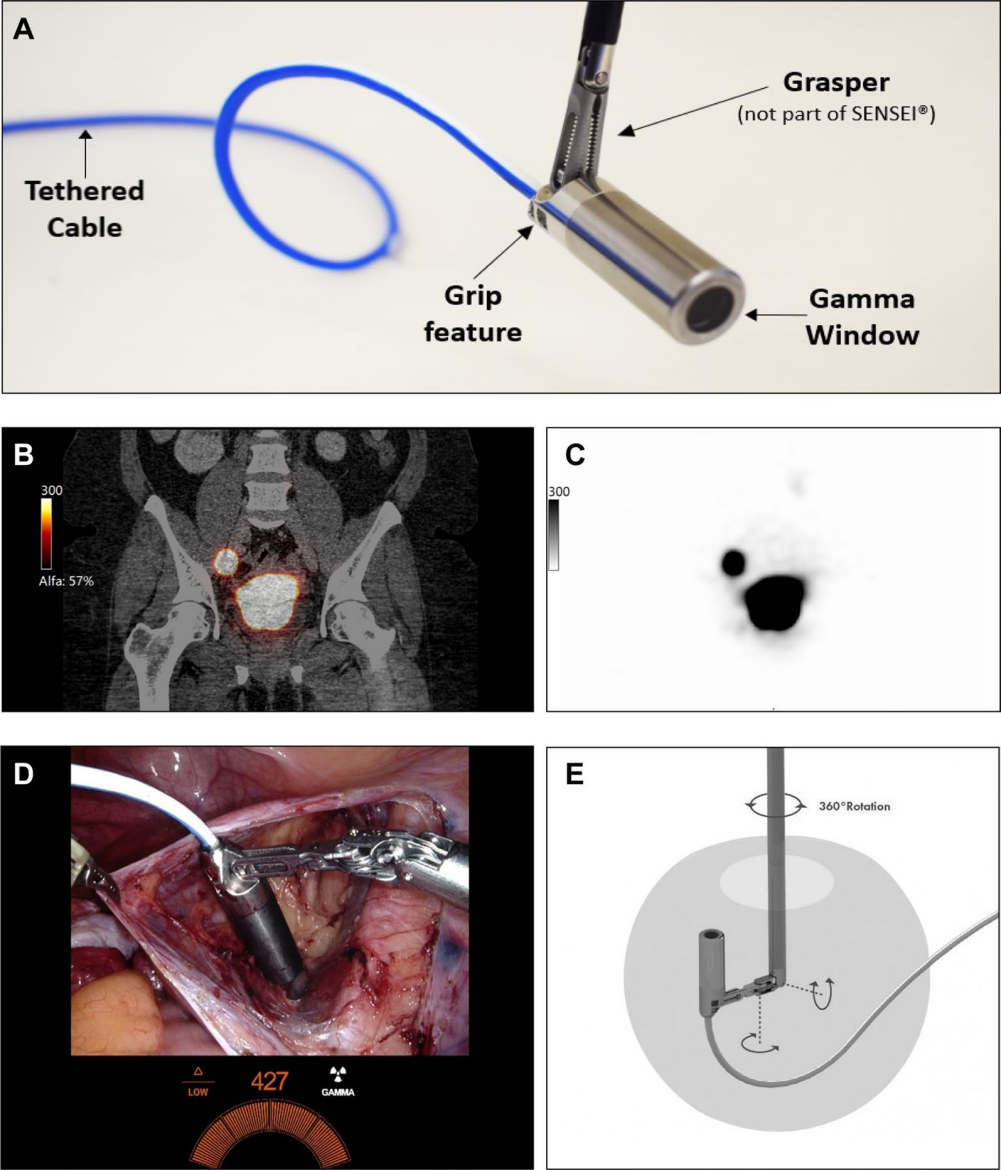
**A** Overview of the tethered drop-in γ-probe. **B** Coronal fusion SPECT/CT image at 90 min postinjection with 240 MBq ^99m^Tc into four quadrants of the cervix. The right SLN is localized in the obturator fossa. **C** Corresponding stand-alone SPECT/CT image. **D** Surgical view of robot-assisted radioguided SLN detection in the right obturator fossa. Integration of the gamma probe signal in the surgical view can be seen in the bottom center. **E** Rotational features of tethered drop-in γ-probe when grasped

For SLN detection the CE-marked SENSEI® laparoscopic tethered drop-in γ-probe system (Lightpoint Medical Ltd, Chesham, UK) was used. The TDIP is 12 mm in diameter, is connected to a control unit by a 3.2 mm cable of 3 m long and is equipped with a grip feature which enables manipulation by multiple (robotic) laparoscopic graspers (Fig. 1). In order to integrate the numerical signal from the TDIP (counts per second – CPS) in the display of the robotic console, the control unit was connected to the robot with a DVI cable (Fig. 1D). The TDIP has undergone comprehensive preclinical testing, which demonstrated proof of concept and safety for its intended use. Before the start of this trial a full Risk Management Review was conducted in accordance with ISO 14971:2012.

After calibration of the TDIP with a Cobalt-57 sealed source at the operation room, which took approximately one minute and could be performed during docking of the robot, the TDIP was introduced using a 12 mm assistant trocar. Subsequently, the TDIP was grasped and maneuvered by the surgeon using the da Vinci Prograsp™ forceps (Intuitive Surgical, Sunnyvale, USA). Time of detection and in vivo count rate of each identified SLN were recorded. Anatomical SLN location was coded according to a pre-specified format also used for SPECT/CT assessment. Sentinel lymph nodes, defined as lymph nodes mapped with SPECT/CT and/or intraoperative radioactivity, were excised based on the surgeon’s discretion. After removal, SLNs were measured ex vivo with the conventional 90 degrees rigid laparoscopic γ-probe (Europrobe 3 Coelioscopique, Euromedical Instruments, Le Chesnay, France). In case of detection failure with TDIP, the rigid γ-probe was used in vivo to assess radioactivity. After excision of SLNs, residual radioactivity of the left and right hemipelvis was carefully measured with TDIP and deemed negative if background count was less than 10% of the highest SLN count in that hemipelvis. Detection of SLNs by radioguidance was attempted in all patients, including those with no SLNs detected on SPECT/CT. All excised SLNs were sent in for pathological ultrastaging. Ultrastaging consisted of step sectioning at multiple levels and immunohistochemical staining with pancytokeratin antibodies, to increase the finding of low volume disease [13]. Surgery proceeded according to the current standard of care as previously described [12, 14]. Intraoperative and postoperative adverse events were scored according to the Common Terminology Criteria for Adverse Events version 5.0 (CTCAE v 5.0) [15].

After the first and after the tenth procedure the surgeons had to fill in a questionnaire in which the usability of TDIP was discussed (see Additional file 2). Statements were scored as: strongly disagree, agree, neither agree or disagree, agree, strongly agree, or do not know.

### Statistical methods

Depending on the data type and its distribution, (non)parametric tests were used as appropriate. For statistical analyses, SPSS statistics (IMB Corp., USA) was used. Statistical tests were two-sided with significance set at *p* < 0.05.

## Results

### Patient characteristics

Table 1 shows the baseline characteristics of included patients. The majority of patients were diagnosed with a squamous cell carcinoma of the cervix FIGO stage IB1 (< 2 cm) or IB2 (2–4 cm). Four patients received postoperative adjuvant treatment indicated by lymph node metastasis (*n* = 2), fulfilling Sedlis criteria (*n* = 1 [16], or positive margins (*n* = 1).

**Table 1:** Baseline characteristics

	Patients (*n* = 10)
Age (years), median (range)	39 (26–72)
BMI (kg/m^2^), median (range)	24.6 (20.3–39.6)
History of abdominal surgery	3
*FIGO stage (2018)*	
IA1 (+ LVSI)	2
IB1	5
IB2	3
*Histology*	
Squamous cell carcinoma	7
Adenocarcinoma	3
*Grade*	
1 – well differentiated	2
2 – moderately differentiated	5
3 – poorly or undifferentiated	3
Lymph vascular space invasion	4
*Surgery performed*	
SLN procedure + PLND + radical hysterectomy	4
SLN procedure + PLND	4
SLN procedure + conisation/hysterectomy	2
Adjuvant treatment	4

*BMI*—body mass index; *FIGO*—International Federation of Gynecology and Obstetrics; *LVSI*—lymph vascular space invasion; *SLN*—sentinel lymph node; *PLND*—pelvic lymph node dissection.

### Sentinel lymph node procedure

Overall and bilateral SLN detection rates on SPECT/CT were 100% (10/10 patients) and 90% (9/10 patients), respectively. In total 22 SLNs were detected on SPECT/CT (see Table 2). With use of TDIP, 21 of these SLNs were identified intraoperatively, resulting in an overall detection rate of 100% and a bilateral detection rate of 80%. The ex vivo detection rates with the rigid laparoscopic γ-probe were identical; the in vivo detection rate was not assessed as the device was only used for ex vivo confirmation. Of the two patients with unilateral SLN detection with TDIP, one patient had unilateral ^99m^Tc uptake only. The other patient presented with an atypical SLN location at the aortic bifurcation that was detected only by SPECT/CT but missed with TDIP intraoperatively. The radioactivity of SLNs, in CPS, measured with TDIP versus rigid γ-probe were not significantly different (median CPS 149 and 117, respectively, *p* = 0.69). A median of 3 SLNs (range 2–4) per patient were excised.

**Table 2:** Radioguided sentinel lymph node detection: outcomes per sentinel lymph node

	SLNs (*n* = 22)
*SLN identified with*	
SPECT/CT	22 (100%)*
Intraoperative ^99m^Tc (drop-in/rigid γ-probe)	21 (95.5%)**
*SLN status*	
Negative	16
Isolated tumor cells	3
Macrometastasis	3

*SLN*—sentinel lymph node; *SPECT/CT*—single-photon emission computed tomography/computed tomography; ^*99m*^*Tc*—technetium-99m nanocolloid

^*^Including one out of template SLN (near aorta bifurcation); not detected with TDIP or rigid gamma probe

^**^Including one out of template SLN (*deep* obturator space); on SPECT/CT classified as within template (i.e., obturator space)

At final histopathology three SLNs showed macrometastasis (in two patients) and three SLNs showed isolated tumor cells (in three patients). One patient with macrometastasis in two SLNs also showed metastasis in two additional non-SLNs obtained at pelvic lymph node dissection (2/21). No false-negative SLNs occurred in patients receiving additional pelvic lymph node dissection (PLND). All metastatic SLNs were detected with the TDIP in vivo and radioactivity of these SLNs was confirmed with the rigid γ-probe ex vivo.

**Fig. 2:**
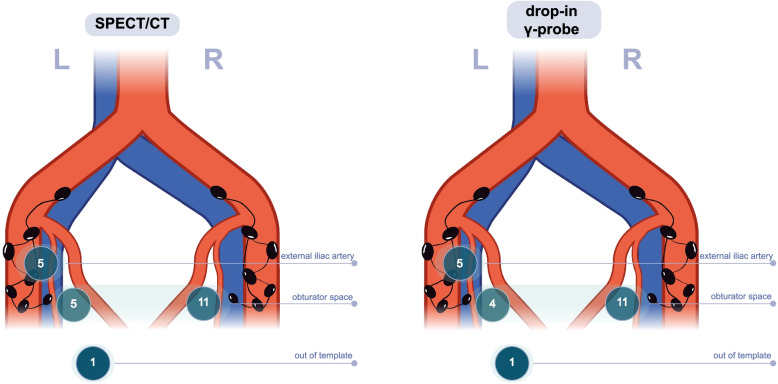
Sentinel lymph node localization with preoperative SPECT/CT (left) and intraoperative drop-in γ-probe (right). The out of template node detected with SPECT/CT only was located near the aortal bifurcation (and not detected with the drop-in γ-probe). The out of template node detected intraoperatively with drop-in probe was located in the deep obturator space (on SPECT/CT assessed as located within the obturator space)

Figure 2 shows the localization of SLNs detected preoperatively with SPECT/CT and intraoperatively with the TDIP. In the right pelvis all SLNs detected with either preoperative or intraoperative radioguidance were located in the obturator space. In the left pelvis TDIP identified five SLNs near the external iliac artery, four SLNs in the obturator space and one SLN in the deep obturator space (i.e., distal from the obturator nerve; classified as out of template). Preoperative SPECT/CT showed similar locations, except for one SLN located near the aortal bifurcation that did not have an intraoperative signal with either γ-probes. The SLN classified as out of template during surgery (identified in the deep obturator space) was on SPECT/CT classified as located within the obturator space.

In one case, TDIP detected two separate SLNs intraoperatively which were reviewed as being one hot node on SPECT/CT. In one case SPECT/CT showed two small hot nodes close to each other, which was intraoperatively detected and excised as one SLN (and confirmed as one SLN at pathology).

No adverse events related to SLN procedure occurred.

### Surgeon questionnaire

Based on surgeon questionnaires, safety (assessed in “clinical performance”), overall ease of use and maneuverability (assessed in “usability function”), and control (“assessed in “usability ergonomics”) of the TDIP system were highly rated. Surgeons agreed on the TDIP potential to replace other tools for SLN detection like rigid laparoscopic γ-probes. Surgeons were asked how many patients they thought would be needed to become confident with using the device. After the first procedure this was thought to be five patients; after the last procedure this number was adjusted to three patients. The questionnaire revealed that the ease of grasping the probe was not uniformly agreed on. Picking up the probe from the pelvis could be challenging without grasping the wire with the robotic forceps or without the bedside assistant lifting the probe from the pelvic tissue by pulling the wire. In all cases the TDIP was grasped successfully.

## Discussion

This is the first prospective study to use a TDIP for detecting SLNs in women with cervical cancer. The TDIP SLN detection rates were similar to the rigid laparoscopic γ-probe. No adverse events related to the SLN procedure or use of the TDIP occurred.

The detection, hardware and electronic principles of TDIP are similar to the rigid laparoscopic γ-probes used in the past decades for minimally invasive SLN procedures, but the TDIP has several distinct advantages over the rigid laparoscopic γ-probe. First, TDIP is typically used together with the robotic tissue grasper. The wristed articulation of this tissue grasper optimizes maneuverability, while control of the probe stays in the hands of the surgeon. A previous non-human study focusing on gripping features of a prototype drop-in γ-probe showed that its use increases the degrees of freedom with which detection can be performed [6]. The improved maneuverability showed to enhance searching the pelvis systematically in every direction and focusing on objective radiation while avoiding high background signal of the injection site. Second, the ability to connect TDIP to an external display (e.g., da Vinci TilePro™) further enabled integration into existing surgical equipment and workflows. Third, because of its thin wire, TDIP can remain in vivo throughout the procedure without preventing other instruments to be inserted through that particular trocar.

Surgeons participating in this study rated TDIP as being easy and intuitive to use, which most likely leads to a short learning curve. This is facilitated by the TDIP’s grip, which is compatible with multiple surgical instruments like tissue graspers, needle holders and Maryland bipolar forceps. The improved maneuverability and autonomous control of the TDIP might shorten the duration of the SLN procedure. A potential disadvantage could be increased costs as the current version of the TDIP used in this study is a disposable. However, no cost-effectiveness comparison of the different probes was performed and a reusable version of the TDIP used in this study is expected this year.

Previous studies in prostate cancer substantiate our findings and point out similar advantages of an autonomously controlled drop-in gamma device. Animal studies and first-in-human translations on the use of different TDIP systems have suggested its usability and led to further improvements in the design of these specific γ-probes [6, 9, 17, 18]. Recent studies in prostate cancer patients undergoing a robot-assisted SLN procedure, showed an increased SLN detection rate with a prototype laparoscopic tethered drop-in γ-probe compared to a conventional rigid γ-probe, which could be attributed to the improved maneuverability and autonomous probe control [18, 19].

Recently, the conventional and well-researched method of SLN detection with ^99m^Tc SPECT/CT is increasingly experiencing competition from nonradioactive alternatives like fluorescence guidance, especially in (robot-assisted) laparoscopic procedures [20–22]. Fluorescence guidance using indocyanine green (ICG) as a lymphatic tracer allows for real-time visualization of lymph nodes during surgery. Other advantages of ICG are its relatively low cost and easy logistics (injected under anesthesia, no preoperative hospital admission required) [22]. One of the disadvantages of ICG is considered to be the lack of preoperative imaging [23]. Research suggests preoperative imaging with ^99m^Tc SPECT/CT can enhance the detection of true SLNs by guiding the surgeon to the right anatomical region, especially when SLNs are present at atypical locations [24, 25] Another disadvantage of ICG is its rapid progression toward second echelons nodes, due to ICG’s small hydrodynamic diameter, undesirably leading to a higher number of (non-)SLNs removed compared to using ^99m^Tc [26, 27]. Until now, studies comparing ICG with the more conventional method of a radiotracer and blue dye lack compelling evidence that fluorescence can safely replace the conventional method [28, 29]. Hybrid modalities integrating the use of a radiotracer, including preoperative imaging, with real-time intraoperative fluorescence imaging could combine the advantages of both methods and are already being studied in other types of cancer [23, 25, 30].

This study had several limitations, notably the small number of participants enrolled. As a pilot study assessing feasibility of a new device, no randomization was implemented between use of the TDIP and the rigid laparoscopic γ-probe in vivo. The SLN mapping was performed by two highly experienced gynecological oncologists specialized in robot-assisted surgery. Thus the learning curve of these surgeons is probably steeper than less experienced colleagues.

## Conclusions

This first-in-women study shows that a drop-in γ-probe is feasible and safe to use for radioguided robot-assisted SLN detection in cervical cancer patients. Based on surgeon questionnaires, autonomy and probe maneuverability were improved compared to the previous experience with conventional rigid laparoscopic γ-probes.

## Supplementary Information

**Additional file 1**: **Fig. S1**. Format SLN stations cervical cancer.

**Additional file 2:** Questionnaire to assess usability of the TDIP.

## Data Availability

The datasets used and analyzed during the current study are available from the corresponding author on reasonable request.

## Abbreviations

BMI: Body mass index
CPS: Counts per second
FIGO: International Federation of Gynecology and Obstetrics
ICG: Indocyanine green
LVSI: Lymph vascular space invasion
PLND: Pelvic lymph node dissection
SLN: Sentinel lymph node
SPECT/CT: Single-photon emission computed tomography/computed tomography
^99m^Tc: Technetium-99m nanocolloid
TDIP: Tethered drop-in γ-probe
