# Supplementary material for: Feasibility of a drop-in γ-probe for radioguided sentinel lymph detection in early-stage cervical cancer
Source: EJNMMI Res. 2022 Jun 20;12:36. doi: 10.1186/s13550-022-00907-w (PMC9209631; doi:10.1186/s13550-022-00907-w)
Supplement: Supplementary file 1 — Additional file 1: Fig. S1. Format SLN stations cervical cancer. [file 13550_2022_907_MOESM1_ESM.pdf]

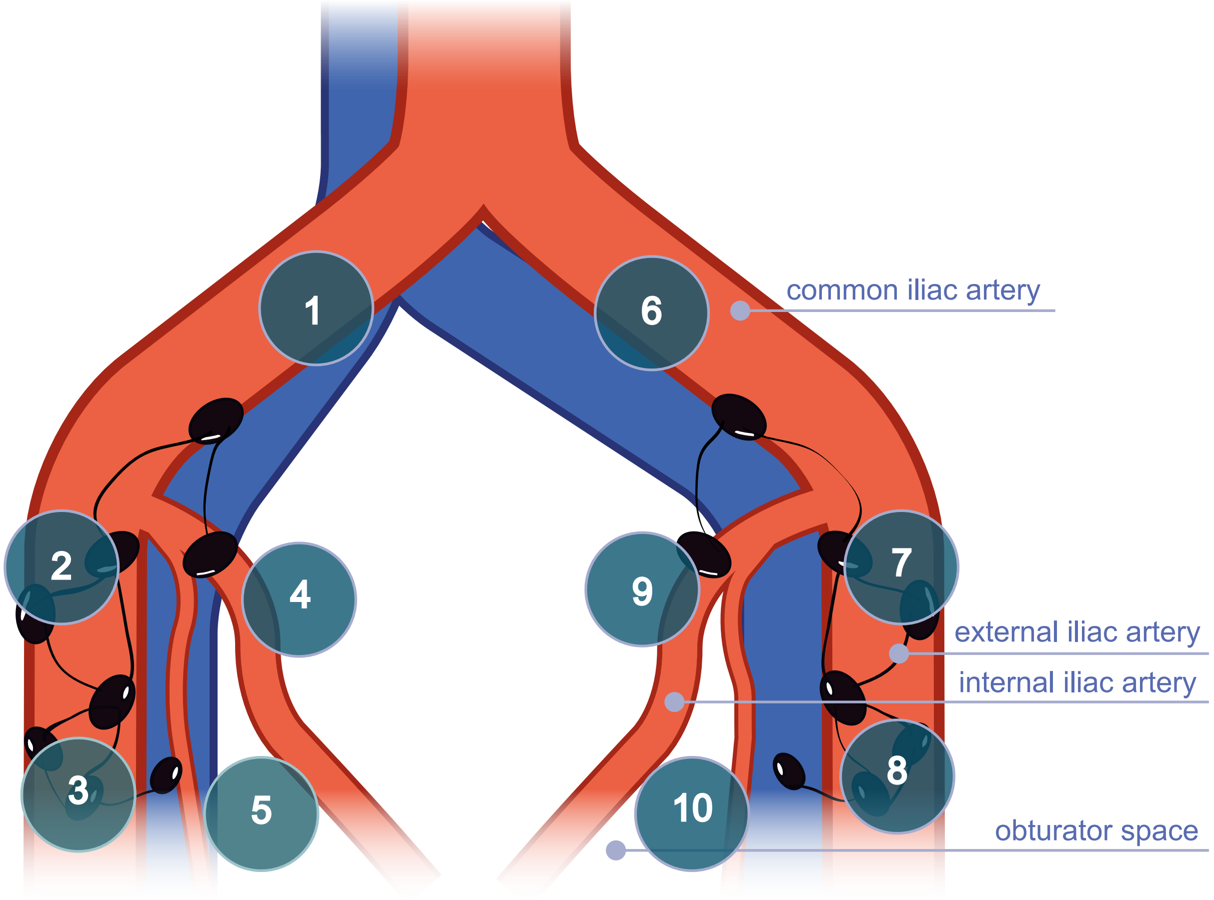

All other locations: out of template

1. Common iliac artery right	6. Common iliac artery left
2. External iliac artery right	7. External iliac artery left
3. Rosenmuller’s node right	8. Rosenmuller’s node left
4. Internal iliac artery right	9. Internal iliac artery left
5. Obturator space right	10. Obturator space left
