# Supplementary material for: Feasibility of a drop-in γ-probe for radioguided sentinel lymph detection in early-stage cervical cancer
Source: EJNMMI Res. 2022 Jun 20;12:36. doi: 10.1186/s13550-022-00907-w (PMC9209631; doi:10.1186/s13550-022-00907-w)
Supplement: Supplementary file 2 — Additional file 2: Questionnaire to assess usability of the TDIP. [file 13550_2022_907_MOESM2_ESM.pdf]

Lightpoint MEDICAL

Study ID ☐-☐☐

Date of Assessment ___/___/______
(dd / mmm / yyyy)

EASE OF USE SURGEON 1/5

SURGERY
(Day 1)

Clinical performance

	Strongly Disagree	Disagree	Neither Agree or Disagree	Agree	Strongly Agree	Do not Know
1. I am happy to use the SENSEI® system intraoperatively to locate sentinel lymph nodes for excision	☐	☐	☐	☐	☐	☐
2. In my opinion the administered activity is sufficient to successfully detect sentinel lymph nodes with SENSEI®	☐	☐	☐	☐	☐	☐
3. In my opinion the time between injection and surgery is sufficient to successfully detect sentinel lymph nodes with SENSEI®	☐	☐	☐	☐	☐	☐
4. The signal from background sources does not impede detection of the sentinel lymph node	☐	☐	☐	☐	☐	☐
5. The field of view of SENSEI® is adequate for detecting sentinel lymph nodes	☐	☐	☐	☐	☐	☐
6. SENSEI® is useful when measuring the residual radioactivity of the surgical site	☐	☐	☐	☐	☐	☐
7. The SENSEI® system has value when used in cervical cancer surgery	☐	☐	☐	☐	☐	☐
8. Based on my experience, I think that the SENSEI® system has the potential to replace other tools used to locate sentinel lymph nodes for excision e.g. rigid gamma probes	☐	☐	☐	☐	☐	☐
9. Pre-operative imaging is a critical element of using the SENSEI® system	☐	☐	☐	☐	☐	☐
10. Based on my experience I think that the SENSEI® system has the potential to be used without pre-operative imaging to locate sentinel lymph nodes for excision	☐	☐	☐	☐	☐	☐

Lightpoint MEDICAL

Subject ID ☐-☐☐

EASE OF USE SURGEON 2/5

SURGERY
(Day 1)

Usability function	Strongly Disagree	Disagree	Neither Agree or Disagree	Agree	Strongly Agree	Do not Know
11. I found the SENSEI® system easy to use	☐	☐	☐	☐	☐	☐
12. Turning the SENSEI® system on/off is easy	☐	☐	☐	☐	☐	☐
13. Conducting the probe check is easy to do	☐	☐	☐	☐	☐	☐
14. Conducting the probe check at the beginning of surgery has not disrupted my or my teams clinical practice	☐	☐	☐	☐	☐	☐
15. I am happy handling the radioactive source during the probe check	☐	☐	☐	☐	☐	☐
16. It is easy to maintain the probes sterility during the probe check	☐	☐	☐	☐	☐	☐
17. The time taken to complete the probe check is acceptable	☐	☐	☐	☐	☐	☐
18. I know where to find the probe check code and how to enter it in the Control Unit	☐	☐	☐	☐	☐	☐
19. Inserting/introducing the SENSEI® Tethered Probe into the patient is easy	☐	☐	☐	☐	☐	☐
20. The stiffness of the cable is adequate for successful manipulation of the Tethered Probe	☐	☐	☐	☐	☐	☐
21. The cable does not obscure the field of view during manipulation of the Tethered Probe	☐	☐	☐	☐	☐	☐
22. I find it easy to interpret the audio signals from the SENSEI® Control Unit to localise a sentinel lymph node	☐	☐	☐	☐	☐	☐
23. I find it easy to interpret the visual signals e.g. the count per second number from the SENSEI® Control Unit to localise a sentinel lymph node	☐	☐	☐	☐	☐	☐
24. I understand why I need to change ranges when using the SENSEI® Tethered Probe	☐	☐	☐	☐	☐	☐

Lightpoint MEDICAL

Subject ID ☐-☐☐

EASE OF USE SURGEON 3/5

SURGERY *(Day 1)*

Usability function

	Strongly Disagree	Disagree	Neither Agree or Disagree	Agree	Strongly Agree	Do not Know
25. I know when the SENSEI® system goes into an over-range state and how to change range	☐	☐	☐	☐	☐	☐
26. Using the SENSEI® Control Unit to connect to an external display is easy	☐	☐	☐	☐	☐	☐
27. I understand why I need to change the visual display from MAX to AVG	☐	☐	☐	☐	☐	☐
28. Setting the displayed count rate to AVG is easy	☐	☐	☐	☐	☐	☐
29. Using the SENSEI® Control Unit to take a AVG at 5s reading is easy	☐	☐	☐	☐	☐	☐
30. Removing the SENSEI® Tethered Probe from the patient is easy	☐	☐	☐	☐	☐	☐
31. I know what type of radiopharmaceutical is compatible with SENSEI®	☐	☐	☐	☐	☐	☐

Usability ergonomics

	Strongly Disagree	Disagree	Neither Agree or Disagree	Agree	Strongly Agree	Do not Know
32. I am able to complete all of the scanning I want to with the SENSEI® system in a timely manner, without disrupting my workflow	☐	☐	☐	☐	☐	☐
33. It is easy to use the SENSEI® Control Unit touchscreen	☐	☐	☐	☐	☐	☐
34. The buttons on the SENSEI® Control Unit are easy to use	☐	☐	☐	☐	☐	☐
35. The icons on the SENSEI® Control Unit touchscreen are easy to understand	☐	☐	☐	☐	☐	☐
36. It is easy to see the SENSEI® Control Unit screen from my normal operating position	☐	☐	☐	☐	☐	☐
37. It is easy to hear the SENSEI® Control Unit screen from my normal operating position with other operating room sounds present	☐	☐	☐	☐	☐	☐

Subject ID ☐-☐☐

EASE OF USE SURGEON 4/5

SURGERY
(Day 1)

38. It is easy to control the volume level of the audio signal using the SENSEI® Control Unit	☐	☐	☐	☐	☐	☐
39. Gripping onto the SENSEI® Tethered Probe is easy	☐	☐	☐	☐	☐	☐
40. Manipulating the SENSEI® Tethered Probe to scan all areas I want to is easy	☐	☐	☐	☐	☐	☐
41. The SENSEI® Tethered Probe is suitable for use in patients of all sizes, e.g. a patient with a narrow pelvis	☐	☐	☐	☐	☐	☐

42. Which of the following areas have you used the SENSEI® Tethered Probe to scan

Left-hand side of the patient	Right-hand side of the patient	
☐ Obturator LN package	☐ Obturator LN package	☐ Pre-sacral LN package
☐ Internal Iliac LN package	☐ Internal Iliac LN package	☐ Common Iliac LN package
☐ External Iliac LN package	☐ External Iliac LN package	

	Strongly Disagree	Disagree	Neither Agree or Disagree	Agree	Strongly Agree	Do not Know
43. It is easy to find a location in the operating theatre for the SENSEI® Control Unit and SENSEI® Tethered Probe	☐	☐	☐	☐	☐	☐
44. It is easy to find a location to store the SENSEI® system	☐	☐	☐	☐	☐	☐

Lightpoint MEDICAL

Subject ID ☐-☐☐

EASE OF USE SURGEON 5/5

SURGERY
(Day 1)

**Training/Supporting documentation**	**Strongly Disagree**	**Disagree**	**Neither Agree or Disagree**	**Agree**	**Strongly Agree**	**Do not Know**
45. The training I received on the SENSEI® system was useful	☐	☐	☐	☐	☐	☐
46. I refer to the instructions for use to find additional information when I need to	☐	☐	☐	☐	☐	☐
47. I refer to the packaging paper insert to find additional information when I need to	☐	☐	☐	☐	☐	☐
48. I refer to the training video to find additional information when I need to	☐	☐	☐	☐	☐	☐

49. Roughly how many patients did you think it needed for you to become confident using the device?

_________ patients
